# Self-Organization of Anastral Spindles by Synergy of Dynamic Instability, Autocatalytic Microtubule Production, and a Spatial Signaling Gradient

**DOI:** 10.1371/journal.pone.0000244

**Published:** 2007-02-28

**Authors:** Thomas Clausen, Katharina Ribbeck

**Affiliations:** European Molecular Biology Laboratory, Heidelberg, Germany; University of California, Davis, United States of America

## Abstract

Assembly of the mitotic spindle is a classic example of macromolecular self-organization. During spindle assembly, microtubules (MTs) accumulate around chromatin. In centrosomal spindles, centrosomes at the spindle poles are the dominating source of MT production. However, many systems assemble anastral spindles, i.e., spindles without centrosomes at the poles. How anastral spindles produce and maintain a high concentration of MTs in the absence of centrosome-catalyzed MT production is unknown. With a combined biochemistry-computer simulation approach, we show that the concerted activity of three components can efficiently concentrate microtubules (MTs) at chromatin: (1) an external stimulus in form of a RanGTP gradient centered on chromatin, (2) a feed-back loop where MTs induce production of new MTs, and (3) continuous re-organization of MT structures by dynamic instability. The mechanism proposed here can generate and maintain a dissipative MT super-structure within a RanGTP gradient.

## Introduction

The mitotic spindle is a MT super-structure which mediates chromosome separation during cell division. One key event during spindle assembly is the accumulation of MTs around chromatin. While centrosomes are considered to be the main source of MT production in mitotic spindle assembly, the mechanism of MT production during assembly of anastral spindles, which do not have centrosomes, is unknown. Anastral spindles assemble during meiosis in, for example, oocytes and egg cells. To further our understanding of the MT production mechanism during anastral spindle assembly, we measured the production rate of spindle MTs. Based on our data we propose that MTs can induce their own production in an autocatalytic manner. With computer simulations we show that a model based on autocatalytic MT production can explain two key principles of spindle self-organization: 1) The emergence of a single coherent MT super-structure around chromatin and 2) the resilience of the MT super-structure toward external perturbations.

In the first step we measured the rate at which MTs are produced during assembly of spindle precursors. As a model system for chromatin-free, anastral spindle assembly we chose the spindle assembly reaction triggered by RanQ69L in *Xenopus* egg extract [1]–[4]. RanQ69L is a constitutively active mutant of the GTPase Ran which is arrested in the GTP-bound form [5]. It releases a set of spindle factors from sequestration by Importins and induces formation of spindle-like structures [6]–[8].

**Figure 1:**
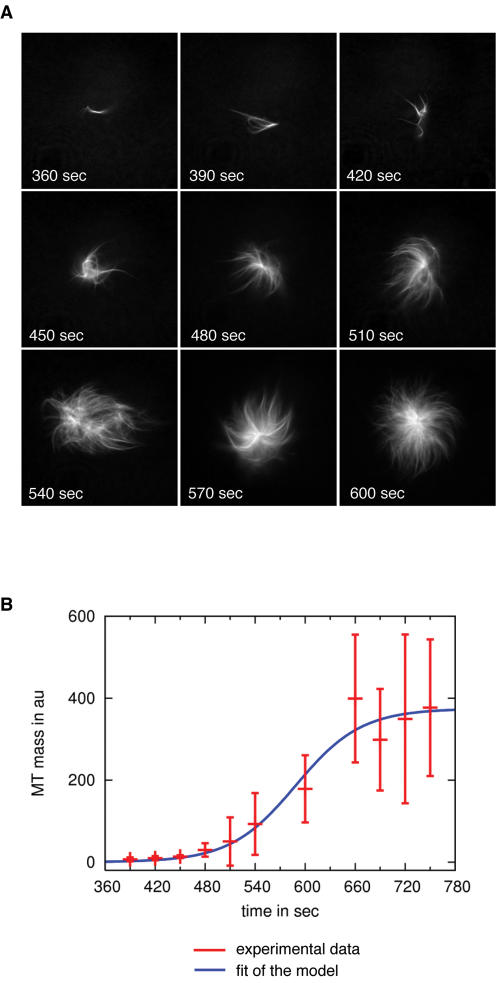
During RanQ69L-mediated aster formation, MTs appear to accumulate exponentially at an initial seed MT. A. Quantification of RanQ69L-mediated MT production in meiotic *Xenopus* egg extract. RanQ69L was added to *Xenopus* extract to initiate the formation of spindle-like structures (asters). After fixation, asters were imaged by wide field microscopy at the indicated time points. The first detectable intermediates of aster formation were few individual MT bundles. Rapidly, more MTs were produced around these seed MTs. The diameter of the asters at 480 sec was 10–30 µm. B. Quantification of MT production mediated by RanQ69L. The plot shows fluorescence of the MTs at the prospective aster (with standard deviations) as a function of time. A logistic model can reproduce the initial exponential rise as well as the saturation of MT mass during aster formation. The logistic model 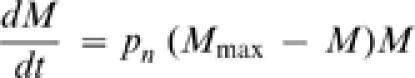 with solution 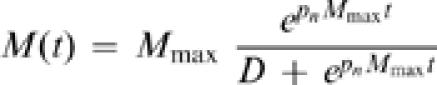 was fit to the experimental data of Figure 1 by minimizing 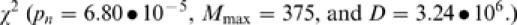 A logistic model supports autocatalytic MT production where MT production is limited by resource depletion.

We added 15 µM RanQ69LGTP to *Xenopus* egg extract and followed spindle pre-cursor assembly by time lapse microscopy (Figure 1A). To quantify MT production we measured the amount of polymerized fluorescent tubulin within emerging MT structures (Figure 1B, see Materials and methods). After 300 sec, a few individual MT bundles were detectable. Shortly thereafter, we observed a rapid local production of MTs at these individual seeds, leading to a high local concentration of MTs within ∼650 sec. Later, the production slowed down and saturation was reached after ∼800 sec. The data can be fit well to an exponential curve at early times up to and including the 660 sec data point (not shown). This indicates that the rate of change of MT production is proportional to the number of existing MTs. The complete data set, including the saturation effect which sets in after ∼800 sec can be fit well by a logistic model which is commonly applied to determine population dynamics (Verhulst-Pearl model [9]–[11], (Figure 1B)). Logistic models consider 1) that the rate of production is proportional to the existing population, and 2) that the rate of production is proportional to the amount of available resources. Thus, population growth is limited by resource depletion. When a population is far from its limits of growth, it grows exponentially, however, when a resource becomes limiting, growth slows down and saturation is reached asymptotically. It is presently unclear which mechanism is responsible for slowing down MT production during spindle assembly.

**Figure 2:**
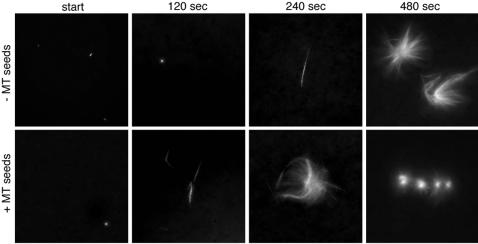
Taxol stabilized MT seeds strongly promote MT production and aster assembly in meiotic *Xenopus* egg extract. The two time series shown here were prepared as in Figure 1A but with addition of soluble Taxol (top series) or Taxol stabilized MT seeds (bottom series). Without MT seeds, the first traces of MTs were visible after ∼240 sec and organized asters (of 10–30 µm diameter) were visible after ∼480 sec. With MT seeds, organized asters were visible already after ∼240 sec. Thus, MT seeds speed up MT production by ∼240 sec. This supports a model where MTs induce the production of more MTs and that this mechanism is responsible for the assembly of MT super-structures.

The exponential and local accumulation of MTs at an initial seed MT (Figure 1A) raises the possibility that MTs stimulate their own production in an autocatalytic manner. If this hypothesis is correct, then it may be possible to stimulate aster formation by providing seed MTs together with RanQ69L to the extract. To test this, we performed the aster assembly reaction in the presence of 25 nM Taxol-stabilized MT seeds (the endogenous tubulin concentration in egg extract is in the range of 20 µM). Together with the solution of Taxol-stabilised MT seeds, we introduced 4 nM of Taxol to the extract (see Materials and methods). To ensure that Taxol at this concentration does not affect spindle formation kinetics we performed the control reaction with Taxol together with RanQ69L. Our data show that in the absence of MT seeds, MT formation progressed as observed in the previous experiment; the first traces of MTs were detectable at ∼240 sec, and organized asters emerged at ∼480 sec (Figure 2). In contrast, in the presence of seeds, many individual MT bundles were detectable already after 120 sec and high local concentrations of MTs were reached already after ∼240 sec. Note that the MT seeds were not labeled with Rhodamine tubulin; all detectable MTs emerged either from new nucleation at the seeds or elongation from the seeds. Importantly, the addition of 25 nM soluble tubulin had no effect on aster formation (not shown). Thus, polymerised tubulin appears to be able to stimulate MT production in egg extract. Next, we quantified MT production in asters in the presence and absence of MT seeds (Table 1). After 240 sec, the MT mass was higher by nearly a factor of 10 when MT seeds were present. After 600 sec the amount of polymerized tubulin was approximately equal in the presence and absence of MT seeds. This result confirms the previous observation that the production of MTs, and thus the assembly of asters, is significantly faster in the presence of MT seeds and suggests that MTs can stimulate the production of new MTs.

**Table 1:** MT production during aster formation around Taxol stabilized MT seeds.

TIME	MT MASS W/O seeds	MT MASS w/ seeds
**240 sec**	6.9+/−4.8 (13)	118+/−97 (19)
**600 sec**	276+/−162 (23)	216+/−246 (24)

Quantification of RanQ69L-mediated MT production in meiotic *Xenopus* egg extract after 240 and 600 sec (MT mass in arbitrary units). The number of sample images used is given in parenthesis. In the right column, Taxol stabilized MT seeds (25 nM) were added together with RanQ69L. With Taxol stabilized MT seeds, aster formation is significantly faster and already after 240 sec the MT mass is close to saturation. This indicates that the MT seeds promote MT production, possible by stimulating autocatalytic MT production.

What is the implication of autocatalytic MT production for spindle assembly? As discussed above, autocatalytic MT production can generate a local high concentration of MTs (Figure 1A, B). In our experiments, where RanQ69L is homogeneously distributed in extract, we observe multiple such MT structures (data not shown, [1]–[4]). This is in contrast to the cell, which forms one and only one MT super-structure around chromatin. A RanGTP gradient has been proposed to provide the spatial cues necessary to position the spindle around chromatin (reviewed in [7], [8], [12]). Like all GTPases, Ran transitions between the GTP and the GDP bound state. The transitions are catalyzed by RCC1 and its direct antagonist RanGAP. RanGAP is a soluble protein and depletes RanGTP from the cytoplasm. In contrast, RCC1 is bound to chromatin and here generates RanGTP. The results are high RanGTP concentrations at chromatin and low RanGTP concentrations at far distance from it [7], [8], [12], [13]. While autocatalytic nucleation can facilitate localized production of MTs, this heterogeneous RanGTP distribution could ensure that a MT super-structure is built at the center of the RanGTP gradient, that is at chromatin.

To investigate the interaction of a heterogeneous RanGTP distribution and autocatalytic MT nucleation, we performed stochastic computer simulations of autocatalytic MT production within a RanGTP gradient. We assumed that a spatially fixed RanGTP gradient extended 50 µm out from a center of highest concentration and that the RanGTP concentration dropped linearly by a factor of three from center to periphery. All MTs in the simulation were static with a length of 5 µm. We initiated the simulation with 200 randomly positioned MTs with an average lifetime of 30 sec to implement dynamic instability [14]–[16]. As MTs disappeared due to dynamic instability, new MTs were produced close to existing MTs with a probability proportional to the RanGTP concentration (see Materials and methods). In these simulations, the number of MTs was kept constant at 200; the exponential increase in the number of MTs was not included in the simulations.

**Figure 3:**
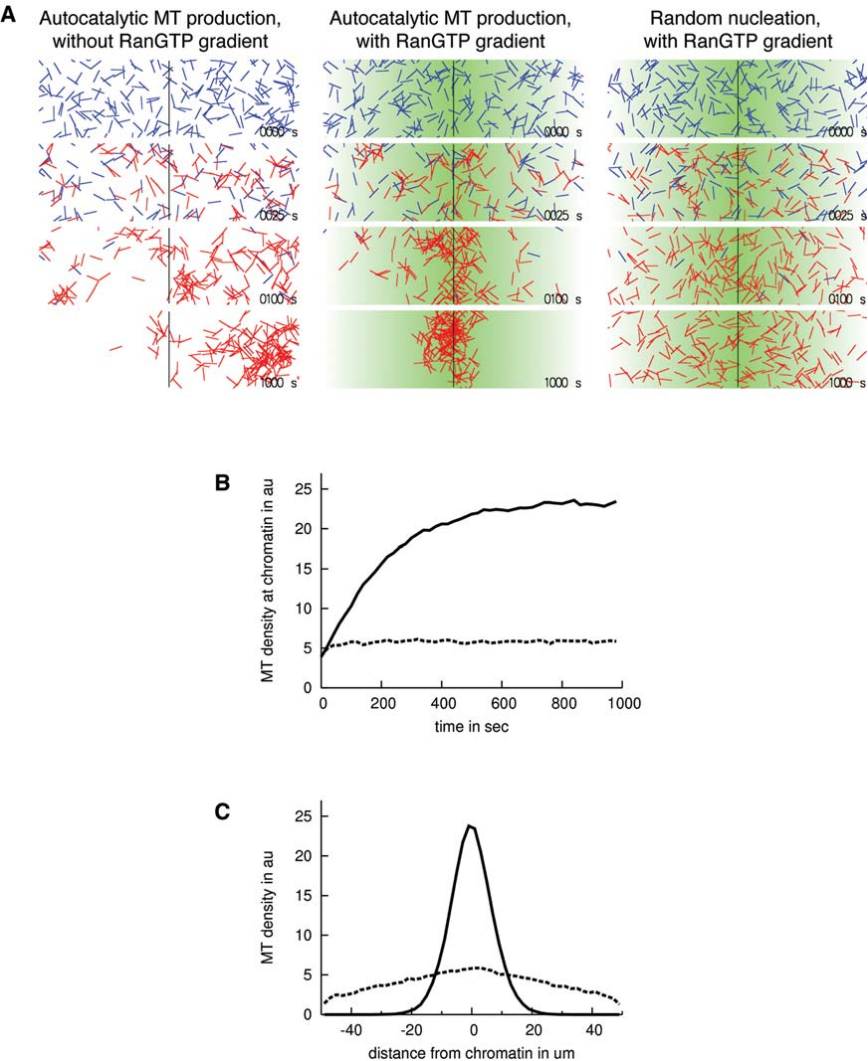
Autocatalytic MT production, dynamic instability, and a RanGTP gradient are necessary and sufficient to target MT production to the center of the gradient. A. MTs in a RanGTP gradient (green indicates high RanGTP concentration). Randomly positioned MTs (blue lines) disappear due to dynamic instability and new MTs are produced (red lines). Without coupling the MT production probability to the RanGTP gradient, MT patches form randomly (left panels). Without autocatalytic MT production, MTs distribute proportional to the RanGTP gradient and do not form patches (right panels). All three components can efficiently concentrate MTs in the center of the gradient (center panels). B. MT density at the centre of the gradient as a function of time with (black line) and without (dashed line) autocatalytic MT production. Without autocatalytic MT production, a constant low MT density is quickly reached, while autocatalytic MT production concentrates MTs in the center of the gradient. C. MT density after 1000 sec as a function of distance from the centre (lines as in B). Autocatalytic MT production efficiently localizes MTs to the center of the gradient.

Figure 3A shows redistribution of MTs over time under three different conditions. First, we simulated the effect of autocatalytic MT production in the absence of a RanGTP gradient (Figure 3A, left). MTs accumulate at random positions in the cytoplasm, a situation reminiscent of chromatin free aster formation in egg extract. Next, we included a RanGTP gradient by increasing the probability of MT production, and thus the actual number of MTs, toward chromatin. Due to dynamic instability, MTs continuously disassemble, which leads to the production of new MTs. If production is independent of existing MTs, new MTs will emerge randomly and distribute proportionally to the RanGTP gradient (Figure 3A, right panels). In contrast, if production is triggered by pre-existing MTs (autocatalytic MT production), new MTs will emerge with higher probability closer to chromatin, simply because more MTs are available toward chromatin (Figure 3A, center panels). By this principle, over several rounds of MT assembly and disassembly, all MTs converge into one coherent super-structure at the position of highest RanGTP concentration. The RanGTP gradient thus provides a small bias, which is amplified by autocatalytic MT production and dynamic instability. The contribution of dynamic instability to MT concentration can be viewed as a form of kinetic proof-reading where spindle structures are continuously re-sampled until one coherent MT super-structure is formed at the position of highest RanGTP concentration [17].

Together, these simulations indicate that autocatalytic MT production and a RanGTP gradient can efficiently target spindle precursors to chromatin if the MTs are dynamically unstable. A combination of all three components efficiently achieves the targeting of MTs to chromatin by eliminating superfluous spindle precursors at distance from chromatin, not by directly targeting MT nucleation to chromatin.

Our simulation experiments reveal that a combination of autocatalytic MT production and dynamic instability can concentrate MTs in the center of a RanGTP gradient disproportionately to the steepness of the RanGTP gradient (Figure 3A, center panels, 3B (black line) and 3C (black line)). We investigated how sensitive the reaction is toward the steepness of the RanGTP gradient. We found that MTs concentrate at the center of the gradient over a wide range of RanGTP gradients (Supplementary Figure S1), illustrating that the concentration mechanism is robust towards fluctuations in the cellular distribution of RanGTP. This means that the shape of the RanGTP gradient is not important for the qualitative effect of MT concentration. This is crucial since cell division needs to commence with extremely high fidelity under varying external conditions. In contrast, for a mechanism where MT production is independent of existing MTs (random nucleation) and where no other non-linear dynamic effects are included, the MT distribution can not exceed the steepness of the RanGTP gradient (Figure 3A, right panels, 3B (dashed line), and 3C (dashed line)).

Our model predicts that the production of MTs in metaphase extract is exponentially sensitive to the concentration of RanGTP (Supplementary Figure S2). Indeed, a dependence of this type has been measured [18]. The measurements show that the number of MTs produced increases exponentially for low RanGTP concentrations and saturates for higher RanGTP concentrations in accordance with the prediction from the model presented here.

**Figure 4:**
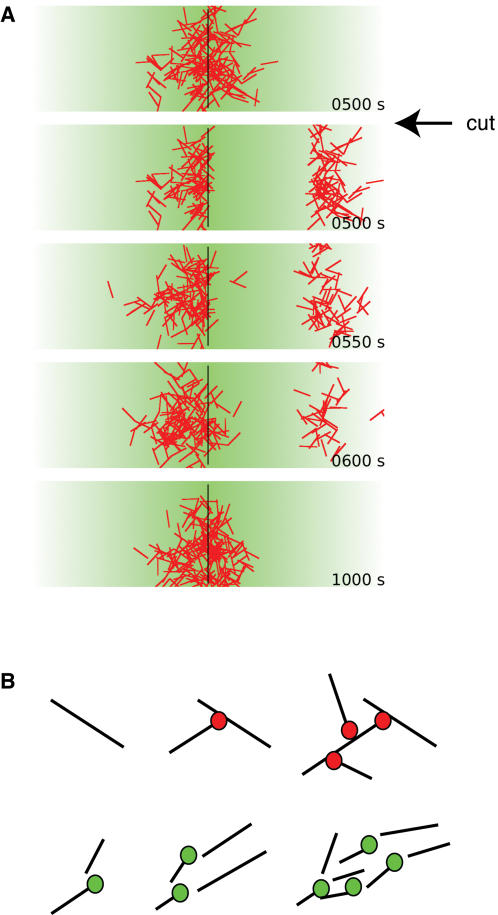
Autocatalytic MT production, dynamic instability, and a RanGTP gradient render MT super-structures robust toward external perturbations. A. The MT super-structure can regenerate within a RanGTP gradient. A fully assembled MT super-structure (top) is divided in two, and one half is moved 20 µm to the right. As MTs disassemble due to dynamic instability, new MTs are produced preferentially in the centre of the gradient. Thereby the severed half disintegrates over time and the integrity of the MT super-structure is re-established. B. Time series of two different mechanisms to achieve autocatalytic MT production. The top series shows a nucleator (red) binding to MTs where it becomes activated and nucleates new MTs. The bottom series shows a MT severing protein (green) binding to MTs and cutting them into fragments. These fragments can grow into new MTs.

The MT super-structure assembled by the mechanism proposed here is self-organizing and dissipative since it constantly consumes energy for MT production. One key characteristic of dissipative self-organizing systems is their capacity to restore themselves. Indeed, as shown by a laser cutting experiment by Tirnauer et al., the spindle has the ability to restore itself even after a substantial part of it has been removed [19]. We asked whether our model has the capacity to regenerate a damaged spindle. We allowed a coherent MT super-structure to form in a RanGTP gradient as described above (Figure 4A, top panel). Then a substantial part of the MT super-structure was removed and placed at a distance from the center of the gradient (Figure 4A, second panel). The simulations revealed that (1) over time the detached MTs disassembled, (2) new MTs were generated at the remaining part of the MT super-structure where the RanGTP concentration was high, and (3) the symmetry of the MT super structure was efficiently re-established (Figure 4A). Importantly, our simulations predict that if any of the three key components, autocatalytic MT production, dynamic instability, or the RanGTP gradient is intervened with, the regeneration mechanism will not work. This could be tested experimentally by combining laser cutting with chemical intervention, for instance by adding MT stabilizing or destabilizing agents such as Taxol or Nocodazole, respectively.

To summarize, our experimental data suggest that the number of MTs increases exponentially during early phases of spindle assembly. We show that this can be reconstituted by a positive feedback mechanism where MTs induce their own production. The positive feedback mechanism is antagonized by dynamic instability, allowing for the disassembly of superfluous or erroneous MT structures. We present a model based on autocatalytic MT production, dynamic instability and a RanGTP gradient around chromatin, which could explain two key principles of spindle self-organization: 1) the emergence of a coherent MT super-structure and 2) the resilience of the spindle toward external perturbations such as severing. Decisively, this model requires the concurrent operation of all three components; in the absence of either autocatalytic MT production, dynamic instability, or a RanGTP gradient, the key features of spindle organization are not achieved. In this context it is important to note that a mechanism analogous to autocatalytic MT production has previously been proposed to contribute to maintaining the spindle [20]. We deduce from our simulations that autocatalytic MT production is indeed one out of three key components necessary for spindle self-organization.

Our model predicts that the disassembly rate of MTs strongly influences the MT accumulation rate at chromatin. The correlation between MT lifetime and accumulation time is simple: Accumulation is the result of several rounds of MT disassembly and re-assembly, and since the duration of each round is given by the MT lifetime, accumulation time is directly proportional to the MT lifetime. Thus, we have the counterintuitive prediction that the more stable MTs are, the longer it takes the spindle to accumulate MTs at the position of highest RanGTP concentration. The implication for the spindle is that altering MT dynamic instability parameters will significantly affect assembly efficiency. This prediction could be tested by perturbing MT lifetime, for example with Nocodazole or Taxol, and measure MT lifetime and spindle assembly times.

By which molecular mechanisms could MTs mediate autocatalytic MT production? Our simulations are consistent with a nucleator that is inactive in solution and becomes activated when it binds to a MT (Figure 4B, top panels). Such a hypothetical nucleator would function analogously to the actin nucleating complex Arp2/3, which becomes activated on binding to an existing actin filament [6]. For MTs, an analogous mechanism has been proposed to form MT super-structures in higher plants [7] and MT bundles in fission yeast [8]; in both systems γ-tubulin plays a central role. However, alternative or additional mechanisms are also possible. For example, the fragmentation of a MT by a MT severing protein such as katanin would result in the exposure of new plus ends which in turn could be elongated (Figure 4B, bottom panels). This mechanism has indeed been suggested to strongly stimulate MT production at meiotic spindles in *C. elegans* oocytes [21]. In both cases MTs would stimulate their own production.

## Materials and Methods

### Quantification of RanQ69L-mediated MT production in *Xenopus* extract

RanQ69L-induced aster formation was monitored in *Xenopus* egg extract arrested in metaphase of meiosis II (cytostatic factor-arrested (CSF) extracts). The extract was prepared according to [22]. Rhodamine tubulin was prepared as described by [23]. For visualization of MTs, Rhodamine tubulin was added to the extract at a final concentration of 0.2 mg/ml.

RanQ69L was prepared as previously described [24]. To initiate aster formation, 15 µM RanQ69L were added to 20 µl egg extract. Aliquots of the reaction were taken every 30 sec, squash fixed [22], and documented by wide field microscopy. For documentation in Figure 1A and 2, the background intensity was adjusted to be the same for all images.

Quantification of MT production was performed in three independent experiments. In each experiment, the signal intensity of 10 individual asters was determined for each time point. To measure signal intensity, each MT structure was placed at the center of a circular region of 20 µm (which is the diameter of an average aster after 10 min). Average background intensity was subtracted from average signal intensity within the circle to give the final intensity of the aster.

To prepare MT seeds, 30 µM soluble tubulin was polymerized in BRB80 buffer [25] in the presence of 1 mM GTP for 10 min at 37C. 5 µM Taxol (Paclitaxel, Sigma) was added and the reaction was further incubated for 10 min at 37C to stabilize the polymerized MTs. Before initiation of the aster assembly reaction, MT seeds were prediluted in Taxol-free BRB80 buffer and added at a final concentration of 25 nM to the extract. The concentration of Taxol transferred to the extract together with the seeds was 4 nM, thus, the control reaction was performed with 4 nM Taxol in the absence of MT seeds.

### Stochastic simulations of MT production within a RanGTP gradient

MTs were modeled as 5 µm static, linear structures randomly oriented in a 100 µm by 30 µm box. Newly produced MTs where positioned in the box as follows. In the random nucleation simulations, one end of the new MT was positioned at a random position inside the box. In the autocatalytic MT production simulations, one end of the new MT was placed at a random position along a random existing MT. If this new position was outside the box then a new random position was chosen. The gradient was modeled by accepting the position for a new MT only if a random number between 0 and 1 was smaller than 
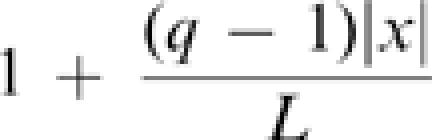
, where 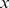 is the position of the new MT with origin in the center of the box, L = 50 µm is the half-width of the box, and q = 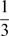 the decrease in MT nucleation probability 50 µm away from the peak of the gradient.

MT disassembly was modeled by removing a MT from the simulation if a random number between 0 and 1 was less than a given time interval times the catastrophe frequency. For all simulations the time interval was 0.1 sec and the catastrophe frequency 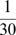 sec^−1^.

To collect statistics for the MT density in Figure 3C, the time from 0 to 1000 sec was divided in 50 intervals and the number of MTs at distance of 1 µm from the box center was counted for each interval for 1000 simulations with 200 MTs. For Figure 3D, the box width (100 µm) was divided into 50 intervals and the number of MTs in each interval was counted for 1000 simulations with 200 MTs after 1000 sec. Increasing the number of MTs in Figure 2 B and C does not change the shape of the curves.

## Supporting Information

Figure S1 The steepness of the RanGTP gradient only weakly affects the efficiency of MT concentration at chromatin. A. Microtubule concentration at chromatin as a function of time for three different slopes of the RanGTP gradient: Reduction to 90% (blue line), 50% (green line), and 10% (red line) at 50 μm from chromatin. B. Steady state spatial distribution of MTs (after 5000 sec) with autocatalytic MT production. Colors as in A. Together, A and B show that increasing the slope of the gradient increases the speed at which MTs are concentrated at chromatin. However, the steady state concentration of MTs at chromatin is reduced only by a factor 2.2 when reducing the slope of the gradient by a factor 9. C. In comparison, if nucleation is independent of existing MTs, then the MTs distribute proportionally to the gradient. Here, a shallow gradient (blue line) results in an almost homogeneous distribution of MTs.
(10.39 MB TIF)

Figure S2 The model presented in this paper predicts an exponential sensitivity of MT mass to the RanGTP concentration. The plot depicts MT mass after 10 minutes as a function of in the logistic equation (see Figure 2). This exponential sensitivity has been measured by [16].
(5.28 MB TIF)
