# Supplementary figures and images for: Self-Organization of Anastral Spindles by Synergy of Dynamic Instability, Autocatalytic Microtubule Production, and a Spatial Signaling Gradient
Source: PLoS One. 2007 Feb 28;2(2):e244. doi: 10.1371/journal.pone.0000244 (PMC1797610; doi:10.1371/journal.pone.0000244)

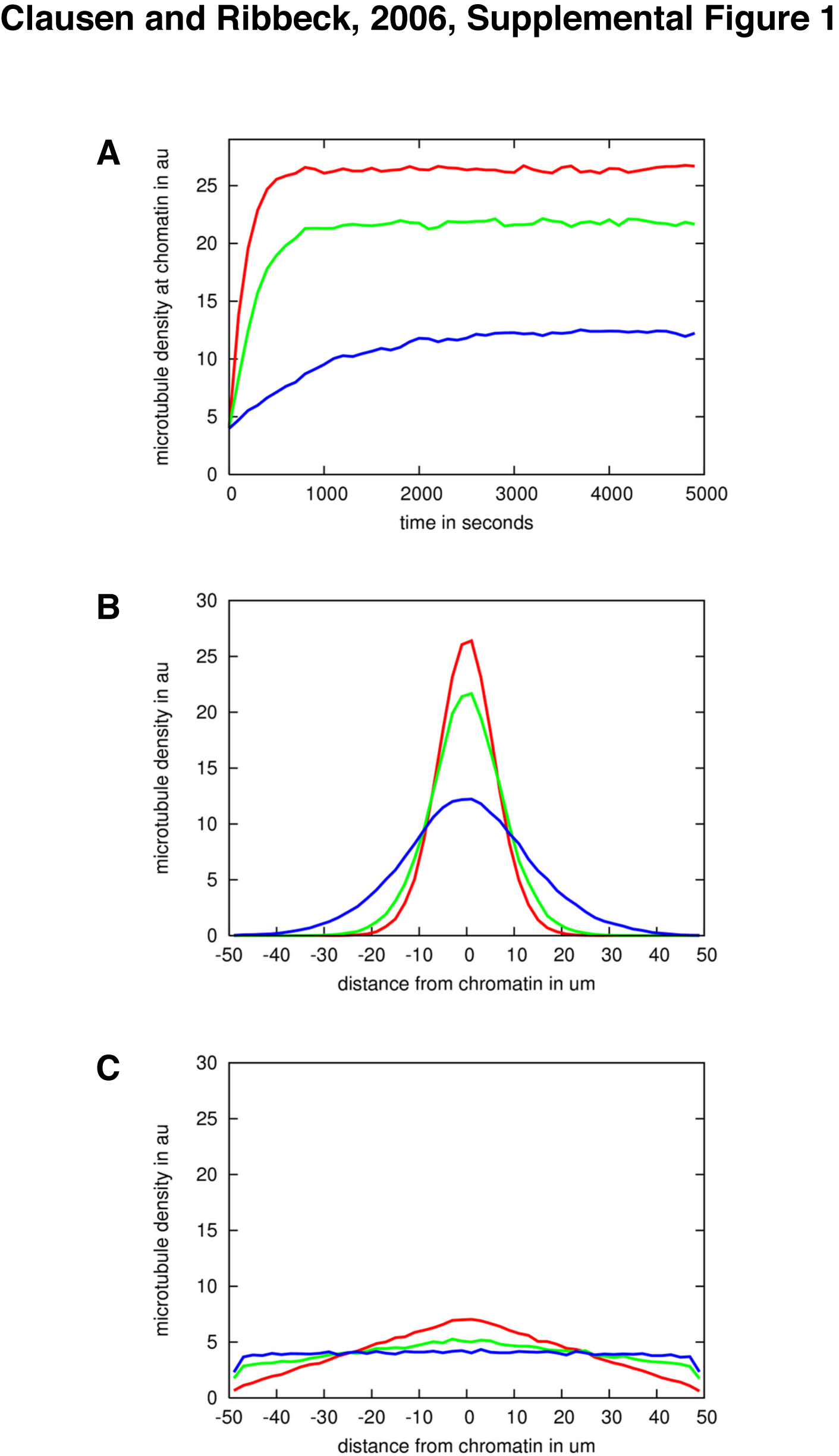

Supplement: Figure S1 — The steepness of the RanGTP gradient only weakly affects the efficiency of MT concentration at chromatin. A. Microtubule concentration at chromatin as a function of time for three different slopes of the RanGTP gradient: Reduction to 90% (blue line), 50% (green line), and 10% (red line) at 50 μm from chromatin. B. Steady state spatial distribution of MTs (after 5000 sec) with autocatalytic MT production. Colors as in A. Together, A and B show that increasing the slope of the gradient increases the speed at which MTs are concentrated at chromatin. However, the steady state concentration of MTs at chromatin is reduced only by a factor 2.2 when reducing the slope of the gradient by a factor 9. C. In comparison, if nucleation is independent of existing MTs, then the MTs distribute proportionally to the gradient. Here, a shallow gradient (blue line) results in an almost homogeneous distribution of MTs. (10.39 MB TIF) [file pone.0000244.s001.tif]

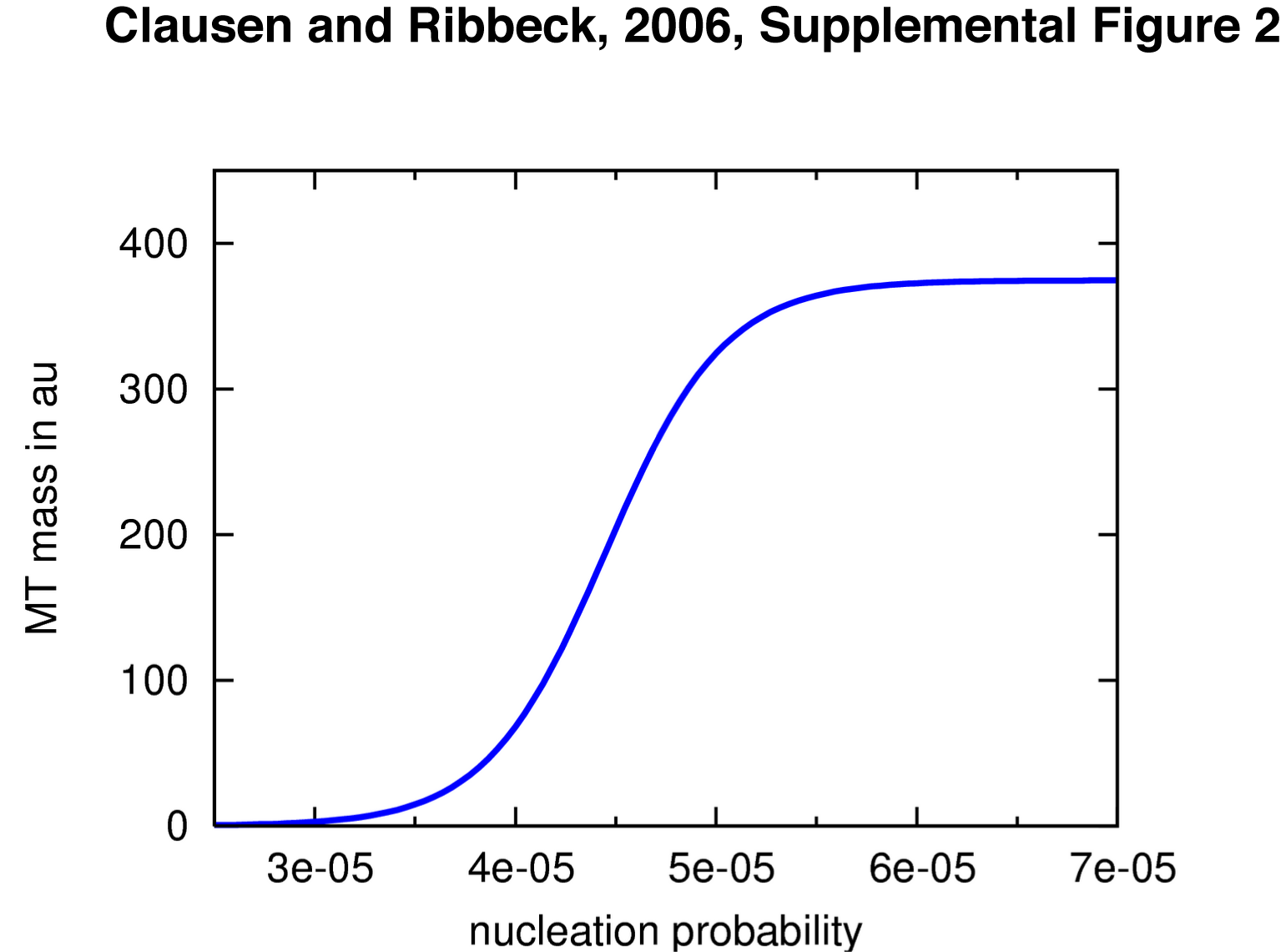

Supplement: Figure S2 — The model presented in this paper predicts an exponential sensitivity of MT mass to the RanGTP concentration. The plot depicts MT mass after 10 minutes as a function of in the logistic equation (see Figure 2). This exponential sensitivity has been measured by [16]. (5.28 MB TIF) [file pone.0000244.s002.tif]
